# Elastic Properties of Magnetorheological Elastomers in a Heterogeneous Uniaxial Magnetic Field

**DOI:** 10.3390/ijms19103045

**Published:** 2018-10-06

**Authors:** Takehito Kikuchi, Yusuke Kobayashi, Mika Kawai, Tetsu Mitsumata

**Affiliations:** 1 Faculty of Science & Technology, Oita University, Oita 870-1192, Japan; 2 Faculty of Engineering, Niigata University, Niigata 950-2181, Japan; t15s811a@mail.cc.niigata-u.ac.jp; 3 Graduate School of Science and Technology, Niigata University, Niigata 950-2181, Japan; mikagoro@eng.niigata-u.ac.jp (M.K.); tetsu@eng.niigata-u.ac.jp (T.M.); 4 ALCA, Japan Science and Technology Agency, Tokyo 102-0076, Japan

**Keywords:** elastomer, magnetorheology, elasticity, modeling

## Abstract

Magnetorheological elastomers (MREs) are stimulus-responsive soft materials that consist of polymeric matrices and magnetic particles. In this study, large-strain response of MREs with 5 vol % of carbonyl iron (CI) particles is experimentally characterized for two different conditions: (1) shear deformation in a uniform magnetic field; and (2), compression in a heterogeneous uniaxial magnetic field. For condition (1), dynamic viscoelastic measurements were performed using a rheometer with a rotor disc and an electric magnet that generated a uniform magnetic field on disc-like material samples. For condition (2), on the other hand, three permanent magnets with different surface flux densities were used to generate a heterogeneous uniaxial magnetic field under cylindrical material samples. The experimental results were mathematically modeled, and the relationship between them was investigated. We also used finite-element method (FEM) software to estimate the uniaxial distributions of the magnetic field in the analyzed MREs for condition (2), and developed mathematical models to describe these phenomena. By using these practicable techniques, we established a simple macroscale model of the elastic properties of MREs under simple compression. We estimated the elastic properties of MREs in the small-strain regime (neo–Hookean model) and in the large-strain regime (Mooney–Rivlin model). The small-strain model explains the experimental results for strains under 5%. On the other hand, the large-strain model explains the experimental results for strains above 10%.

## 1. Introduction

Magnetorheological elastomers (MREs) are soft materials with rheological properties that change with magnetic fields (MF). This effect is called the MR effect. MREs consist of polymeric matrices and magnetic particles. A few decades ago, MREs exhibited MF-induced changes in their elastic modulus which could reach well over ten percent at low strains [1,2]. However, in recent years, several researchers have developed MREs that exhibit hundred-fold changes in their dynamic modulus at low strains [3], or several-fold changes at high strains [4]. Moreover, Mitsumata et al., [5] developed a polyurethane-based MRE with a volume fraction of 0.29 carbonyl iron (CI) particles, that demonstrated drastic and reversible changes in dynamic modulus, more than 200-fold the original value, at high strains. In addition, the effect of a weak MF on vibration transmissibility was investigated for this MRE, for various volume fractions of magnetic particles [6]. Vibration damping is one of the promising applications of MREs [2]. In addition, porous magnetic materials [7] are promising candidates for vibration control and other real-world applications.

In the above-mentioned research, a homogeneous MF was applied to MREs by using well-designed electromagnets (EMs). However, it is difficult to ensure MF homogeneity in real-world applications. In non-uniform MFs, the mechanical properties of MREs also become non-uniform. For example, Varga et al. [8], investigated the direction-dependent MR effect of MREs. Microscale models of the mechanical properties of MREs are discussed in References [9,10,11,12,13,14,15]. Such models are especially useful when designing materials. However, in real-world applications, macroscale mechanical models are also useful for mechanical design. For example, large-strain behavior of MREs under uniaxial compression and tension was experimentally investigated by Schubert et al., [4]. However, a mathematical model of such a macroscale environment has not yet been developed.

**Figure 1:**
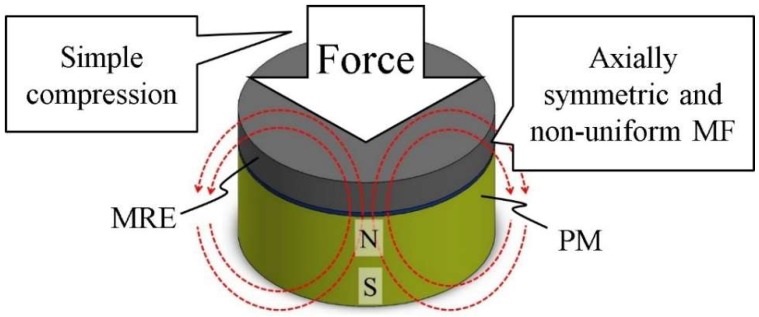
The study system. MRE—magnetorheological elastomer; MF—magnetic field; PM—permanent magnet. Dashed red curves show the MF lines. The MF is axial symmetric, but non-uniform in the vertical direction.

This study aims to establish a simple macroscale model of the elastic properties of MREs under simple compression, for the condition that is schematically shown in Figure 1. We used permanent magnets (PM) to generate the MF. The MRE samples were located on top of the PMs, and a uniaxial but non-uniform MF was applied to them. In this study, the large-strain behavior of MREs with 5 vol % of CI particles was experimentally characterized for two different conditions: (1) shear deformation in a uniform magnetic field; and (2), compression in a non-uniform uniaxial magnetic field. For condition (1), dynamic viscoelastic measurements were performed using a rheometer with a rotor disc and an electric magnet that generated a uniform magnetic field on disc-like material samples. For condition (2), on the other hand, a permanent magnet was used to apply a non-uniform uniaxial magnetic field under cylindrical material samples. We also used finite-element method (FEM) software to estimate the uniaxial distributions of the MF in MREs for condition (2), and designed mathematical models to describe these phenomena. Using these practicable techniques, we estimated the elastic properties of the analyzed MREs for the condition described in Figure 1. 

## 2. Results

### 2.1. Shear Modulus Measurements for Uniform MF

**Figure 2:**
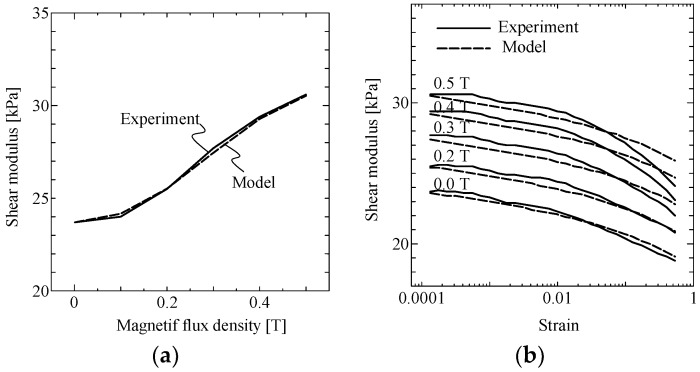
MR effects of MRE in uniform MF. (**a**) MF dependence of shear modulus for small strain (0.01%), and (**b**) strain dependence of shear modulus, for different MF intensities.

Figure 2a shows the MF intensity dependence of shear modulus, for the analyzed MRE, for a small strain of 0.01%. The solid curve shows the experimental results while the dashed curve shows the results for the model that is described in Section 3.1. The curve starts at 23.7 kPa and exhibits an S-shaped increase with increasing magnetic flux density. Although this curve has not reached saturation, we assumed the saturation value was 33 kPa by fitting the curve of Equation (2) shown in Section 3.1. Figure 2b shows the strain dependence of shear modulus, for the analyzed MRE, for shear strains in the 0.01–50% range, and MF intensity in the 0.0–0.5 T range. The difference between the shear moduli for small and large strain regimes is ~5–6 kPa for every analyzed value of MF, including the “off” state.

### 2.2. MF Simulation for Non-Uniform MF

**Figure 3:**
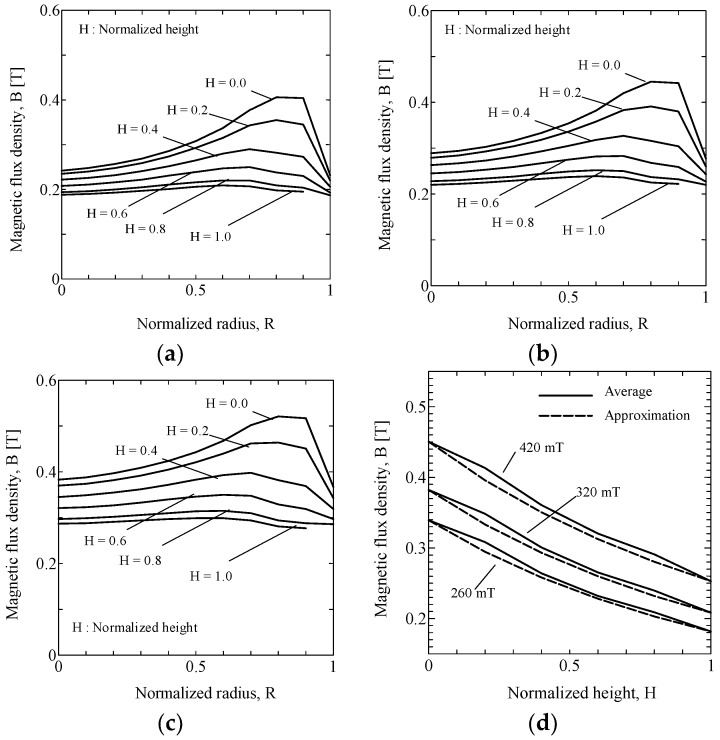
Non-uniform MFs in MREs with PMs of: (**a**) 260 mT; (**b**) 320 mT; (**c**) 420 mT; (**d**) averaged MF distributions for each PM.

The MF distributions for non-uniform MF, shown in Figure 1, were simulated using finite element method (FEM) software. The MF simulation results for three different PMs are shown in Figure 3a–c. The radii and the heights in these figures are normalized with respect to the radii and heights of the PMs, respectively. The horizontal axes, *R*, show the normalized radius, and zero corresponds to the center line of the PMs. The vertical axes show the magnetic flux density, *B*. As shown in these figures, the distributions of MFs are not constant for the same height. The height, *H*, is the normalized height, and zero corresponds to the level of the magnet, while one corresponds to the top surface of the MREs.

### 2.3. Compression Tests for Non-Uniform MF

**Figure 4:**
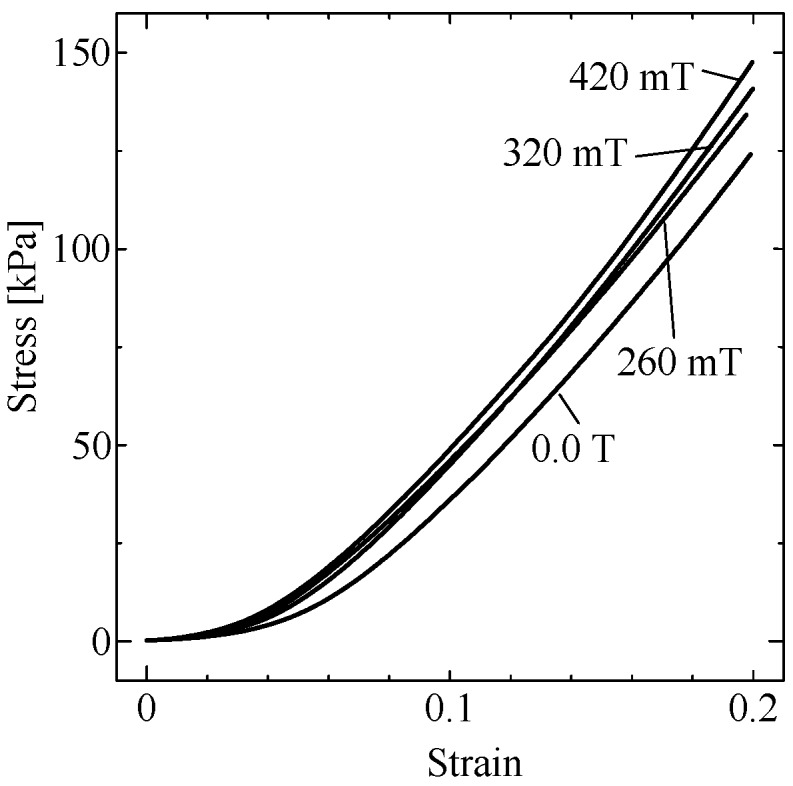
Stress-strain curve for MREs. Both stress and strain represent “engineering” stress and strain. Positive strains correspond to compression strains.

Figure 4 shows the stress-strain curve for MREs under the condition in Figure 1. Positive strains correspond to compression strains. Both stress and strain represent “engineering” stress and strain. The goal of this study was to estimate the “on” state curve of this figure from the “off” state curve and the previous model function.

## 3. Discussion

### 3.1. Shear Modulus Measurements for Uniform MF

The Langevin function [1,16] (Equation (1)) has been widely used in mathematical models, to describe saturation curves of quantities for materials in MFs.

(1) $$\;L\left(x\right)=\coth\left(x\right)-\frac{1}{x}\;$$

Therefore, we modeled the MF dependence of the shear modulus in the small strain regime (<0.01%), G, using the Langevin function (Equation (2)). In this equation, the squared B; magnetic flux density was only selected as an independent variable:

(2) $$\;G\left({\left|B\right|}^{2}\right)=G_{0}+L\left(c_{1}{\left|B\right|}^{2}\right)\cdot \left(G_{\infty }-G_{0}\right)\;$$

where $G_{0}$ and $G_{\infty }$ are 23.7 kPa and 33 kPa, respectively. The value of the fit parameter $c_{1}$ that minimized the modeling error was 15. The modeling result is shown as the dashed line in Figure 2a. This model captures the MF dependence of elasticity for small strains well.

We modified the Kraus model [17] for the strain dependence of the elastic modulus of MREs, as given by Equation (3).

(3) $$\;G\left({\left|B\right|}^{2},\;\gamma \right)=G_{0}+L\left(c_{1}{\left|B\right|}^{2}\right)\cdot \left(G_{\infty }-G_{0}\right)-c_{2}\frac{\left(\frac{\gamma }{\gamma_{0}}\right)-1}{{\left(\frac{\gamma }{\gamma_{0}}\right)}^{\beta }+1}\cdot \Delta G_{\gamma }\;$$

In the above equation, the nominal strain, $\gamma_{0}$, is 0.01%; the strain-related difference between moduli, $\Delta G_{\gamma }$, is 5.4 kPa; the initial modulus, $G_{0}$, is 23.7 kPa; and the saturated modulus, $G_{\infty }$, is 33.0 kPa. The values of the fit parameters, $c_{2}$ and β that, minimized the modeling error were 0.1 and 0.75, respectively. The modeling results are also shown as the dashed lines in Figure 2b. The modeling error is small in the low MF region (<0.3 T) or low strain region (<1%). However, it becomes non-negligible in the high MF and strain region up to 1.8 kPa, which is 7.5% of the initial modulus, $G_{0}$. A single sample was used for a series of tests. The cycle of the tests was conducted from low to high MF and strain. History dependence with the loading cycle appears to be one of the reasons for this modeling error.

### 3.2. MF Simulation for Non-Uniform MF

We can define the MF distribution as a function of *R* and *H*; *B*(*R*, *H*). In this study, we focused on the simple compression mode. Therefore, we can simplify the distribution *B* as a function of only *H*, as in Equation (4). For this purpose, we calculated the field with the weighted mean of the radius:

(4) $$\;\bar{B}\left(H\right)=\overset{N-1}{\underset{i=0}{\sum }}\left\{\left(R_{i+1}^{2}-R_{i}^{2}\right)B\left(\frac{R_{i+1}+R_{i}}{2},\;H\right)\;\right\}\;$$

where $R_{i}$ is the *i*-th normalized radius in the FEM system, and the index runs from the center (*i* = 0) to the outer edge (*i* = *N*) of the PMs.

The averaged MFs, $\bar{B}$, are shown as the solid curves in Figure 3d. These are hyperbolic curves; therefore, we modeled the averaged MF distributions as shown in Equation (5):

(5) $$\;\tilde{B}\left(H\right)=\frac{a}{{\left(H+b\right)}^{2}}\;$$

Here, *a* and *b* are the fit parameters, and the values of these obtained that minimized the modeling error were 2.73, 2.53 for 260 mT, 2.82, 3.04 for 320 mT, and 2.99, 4.03 for 420 mT, respectively. The modeling results are also shown as the dashed curves in Figure 3d. 

### 3.3. Compression Tests for Non-Uniform MF

In general, the compression ratio, λ (=1−ϵ), is used in the mechanical models of elastomers instead of the engineering strain. According to Kuhn’s rubber-like elastic theory, a simple compression of an incompressible neo-Hookean material [18] can be described as follows:

(6) $$\;\sigma =G(\lambda -\lambda^{-2})\;$$

In a general case, the shear modulus, *G*, is defined as in Equation (3). Here, we use the approximated MF (Equation (5)) for *G*. In addition, for simple compression, we assume that the shear strain is very small and equal to $\gamma_{0}$. Then, *G* is simplified as follows:

(7) $$\;G\left({\tilde{B}}^{2}\left(\lambda \right),\;\gamma_{0}\right)\equiv \tilde{G}\left({\tilde{B}}^{2}\left(\lambda \right)\right)=G_{0}+L\left(c_{1}{\tilde{B}}^{2}\left(\lambda \right)\right)\cdot \left(G_{\infty }-G_{0}\right)\;$$

**Figure 5:**
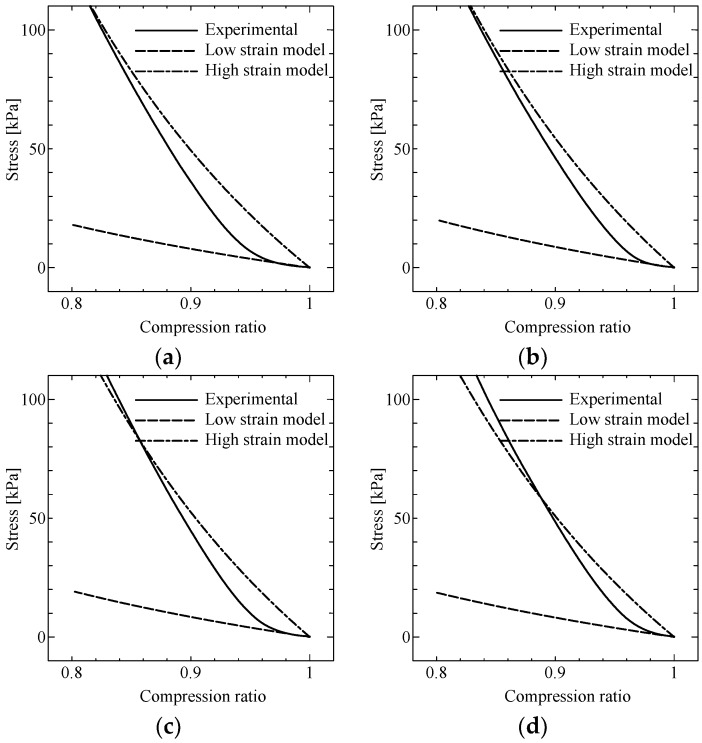
Model of the stress curve for non-uniform MF, for different PMs: (**a**) 0 mT; (**b**) 260 mT; (**c**) 320 mT; (**d**) 420 mT.

We added the Mooney–Rivlin representation [18] to Equation (6) to represent material inhomogeneity, and substituted Equation (7) to obtain the following:

(8) $$\;\sigma =\tilde{G}\left({\tilde{B}}^{2}\left(\lambda \right)\right)\cdot \left(1+c_{3}\lambda^{-1}\right)\cdot \left(\lambda -\lambda^{-2}\right)\;$$

where the elastic modulus is$\;G_{0}$ for the case without a magnet. The value of the fit parameter$,\;c_{3},$ that minimized the modeling error was 0.0 for the low-strain regime (low strain model), and 4.7 for the high-strain regime (high strain model). The experimental results and the modeling results are shown as the dashed curves in Figure 5a–d. The small-strain model (the neo-Hookean model) explained the experimental curves for strains under 5%. On the other hand, the Mooney–Rivlin model explained the experimental curves for strains above 10%. The modeling error was larger for stronger MFs. Material anisotropy of MRE [8] can possibly explain this error.

## 4. Materials and Methods

### 4.1. Synthesis of Magnetorheological Elastomers

Polyurethane elastomers and magnetic elastomers were synthesized using a prepolymer method. Polypropylene glycols (Mw = 2000, 3000), prepolymer cross-linked by tolylene diisocyanate (Wako Pure Chemical Industries. Ltd., Osaka, Japan), dioctyl phthalate (DOP, Wako Pure Chemical Industries. Ltd.), and carbonyl iron (CS Grade BASF SE., Ludwigshafen am Rhein, Germany) particles were mixed using a mechanical mixer, for several minutes. The molar ratio of –NCO to –OH group for the prepolymer was constant, at 2.01 (=[NCO]/[OH]). The median diameter of carbonyl iron particles was 7.0 ± 0.2 μm, determined using a particle size analyzer (SALD-2200, Shimadzu Co., Ltd., Kyoto, Japan). The saturation magnetization for carbonyl iron particles was evaluated to be 245 emu/g, using a SQUID magnetometer (MPMS, Quantum Design Inc., San Diego, CA, USA). 

The mixed liquid was poured into a silicon mold and cured on a hot plate for 60 min at 100 °C. The weight concentration of DOP to the matrix without magnetic particles was fixed at 28 wt %. The volume fraction of the magnetic particles was maintained at 0.05. We synthesized a sheet-shaped MRE with thicknesses of 1.5 mm and 5 mm for shear modulus measurements and compression tests, respectively. Samples were hollowed out by using hollow punches with diameters of 20 mm and 35 mm, respectively. The measured relative permeability of the MRE was 1.22. This value was used in the MF simulations.

### 4.2. Shear Modulus Measurements for Uniform MF

The strain dependence of dynamic modulus was assessed using a rheometer (MCR301, Anton Paar Pty. Ltd., Graz, Austria) at 20 °C. The frequency was constant at 1 Hz. The sample was a disk 20 mm in diameter and 1.5 mm thick. The homogeneous and vertical magnetic field of up to 0.5 T was generated by an EM. 

### 4.3. Compression Tests for Non-Uniform MF

**Figure 6:**
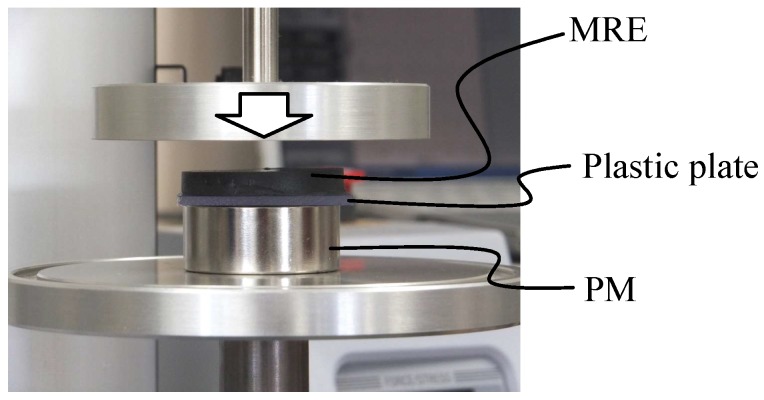
Compression apparatus and setup for a specimen. Three different magnets with the same diameter were used.

A compression apparatus (EZ-SX, Shimazu, Japan) was used at 20 °C for modeling the stress-strain curve of the MRE in the “off” state and for the estimation of the curve in the “on” state. The compression speed was constant at 4.0 mm/min. The MRE sample was a disk 35 mm in diameter and 5 mm thick. The geometrical information is shown in Figure 6. Three types of PMs were used for generating the MF (surface flux density at the center: 260 mT, 320 mT, and 420 mT). The magnetic field strength was measured by a Hall sensor (TM-601, Kanetec Co. Ltd., Ueda, Japan). The common diameter of the magnets was 35 mm. A non-magnetic plastic plate 35 mm in diameter and 0.65 mm thick was inserted between the magnets and MRE samples for easier handling. 

### 4.4. MF Simulation in Non-Uniform MF

**Figure 7:**
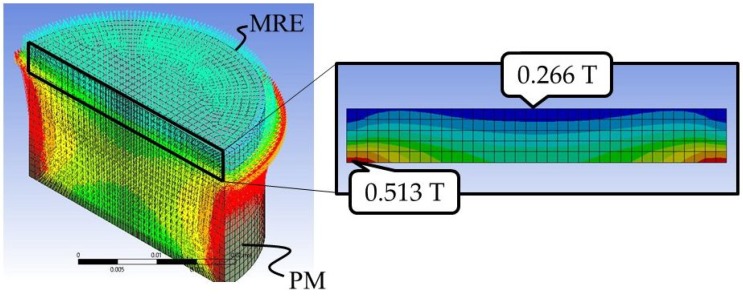
Result of the finite-element method (FEM) analysis. **Left:** vector field of the magnetic flux density. The front surface shows a half-cut surface. **Right:** contour view of the absolute value of the flux density on the cut surface of the MRE. The bottom line represents the contact surface with the magnet via a plastic plate. These figures show the MF is axially symmetric, but non-uniform in the vertical direction.

The finite element method (FEM) was used to estimate the MF distribution in the case of Figure 6. We used FEM analysis software, ANSYS ver.19. Figure 7 shows the cross-section of the MRE and the distribution of magnetic flux density in the cut plane. The distribution varies in the three-dimensional space, but has axial symmetry. To simulate the real MF at the center and top surface of magnets, we set the magnet parameters as shown in Table 1.

## 5. Conclusions

We developed a simple macroscale model of the elastic properties of MREs under simple compression, and in a non-uniform and uniaxial MF. In this study, the large-strain behavior of the MREs with 5 vol % of CI particles was experimentally characterized for two different conditions: (1) shear deformation in a uniform magnetic field, and (2) compression in a non-uniform uniaxial magnetic field. We also used FEM software to estimate the uniaxial distributions of the MFs in the analyzed MREs for condition (2), and developed mathematical models to describe these phenomena. By using these practicable techniques, we estimated the elastic properties of MREs for the small-strain regime (neo-Hookean model) and for the large-strain regime (Mooney–Rivlin model). The small-strain model fitted the experimental curves for strains under 5%. On the other hand, the large-strain model fitted the experimental curves for strains above 10%. 

## Figures and Tables

**Table 1:** Magnet parameters in the FEM analysis.

Flux Density in the Center (mT)	Coercivity (A/m)	Remanent Flux Density (mT)	Thickness (mm)
260	9.07 × 10^5^	1250	8
320	9.07 × 10^5^	1250	10
420	9.10 × 10^5^	1300	15
